# Major Depressive Symptoms Increase 3-Year Mortality Rate in Patients with Mild Dementia

**DOI:** 10.1155/2017/7482094

**Published:** 2017-04-06

**Authors:** Jindong Ding Petersen, Frans Boch Waldorff, Volkert Dirk Siersma, Thien Kieu Thi Phung, Anna Carina Klara Magdalena Bebe, Gunhild Waldemar

**Affiliations:** ^1^Research Unit for General Practice, Department of Public Health, University of Southern Denmark, J.B. Winsløws Vej 9, 5000 Odense C, Denmark; ^2^Department of Mental Health, Mental Health Services in the Region of Southern Denmark, Skovbakken 2, 6000 Kolding, Denmark; ^3^Danish Dementia Research Centre, Department of Neurology, Rigshospitalet, University of Copenhagen, Copenhagen, Denmark; ^4^Research Unit for General Practice and Section of General Practice, Department of Public Health, University of Copenhagen, Øster Farimagsgade 5, P.O. Box 2099, 1014 Copenhagen K, Denmark

## Abstract

Depression and dementia are commonly concurrent and are both associated with increased mortality among older people. However, little is known about whether home-dwelling patients newly diagnosed with mild dementia coexisting with depressive symptoms have excess mortality. We conducted a post hoc analysis based on data from the Danish Alzheimer's Intervention Study of 330 individuals who were diagnosed with mild dementia within the past 12 months. Thirty-four patients were identified with major depressive symptoms (MD-S) at baseline. During the 3-year follow-up period, 56 patients died, and, among them, 12 were with MD-S at baseline. Multivariable analysis adjusting for the potential confounders (age, sex, smoking status, alcohol consumption, education, BMI, household status, MMSE, CCI, QoL-AD, NPIQ, ADSC-ADL, medication, and RCT allocation) showed that patients with MD-S had a 2.5-fold higher mortality as compared to the patients without or with only few depressive symptoms. Our result revealed that depression is possibly associated with increased mortality in patients with mild dementia. Given that depression is treatable, screening for depression and treatment of depression can be important already in the earliest stage of dementia to reduce mortality.

## 1. Introduction

Dementia is a common and devastating chronic neurodegenerative disorder among older people [1]. It is characterized by progressive memory loss, impairments in orientation and concentration, difficulty in language and executive functions, and deficits in spatial awareness, comprehension, and judgment, which all have physical, emotional, and financial impacts on patients and their caregivers [2]. Alzheimer's disease (AD) is the most common etiology for dementia, accounting for 60–80% of all dementia cases [3]. Worldwide, over 46 million people live with dementia, and this number is expected to increase to over 132 million by 2050 [4].

Depression is common in people with dementia, occurring with a prevalence ranging between 10% and 62% [5]. Among AD patients, about 50% may experience a depressive episode at least once during the course of the disease [6]. Apart from other potential determinants, dementia-related biological, behavioural, and physiological changes may play a role in the subsequent development of depression [7–9]. Conversely, among other biological mechanisms, depression triggers the release of stress hormones, and these may cause hippocampal injury of the brain, which may subsequently lead to AD and other forms of dementia [10, 11].

Persons suffering from depression or dementia have increased mortality compared to the general population [12–15]. A recently published meta-analysis investigating mortality in mental disorders including 43 studies of depression showed that the mortality was almost two times higher in people with depression as compared to the general population [13]. Similarly, dementia has been associated with increased mortality from another meta-analysis [16].

Severity and time since disease onset affect mortality in patients with depression or dementia [17–20]. Previous studies have suggested that patients with coexisting depression and severe dementia have higher mortality rate than patients with less severe cognitive impairment [21, 22]. Other studies have also indicated that patients with dementia and severe depression have increased mortality compared to those with less severe depression [23].

However, previous studies have mainly focused on clinical depression among patients with moderate to severe dementia residing in nursing homes [24–26]. To date, little research has been done to investigate mortality among home-dwelling patients with mild dementia coexisting with depressive symptoms.

Thanks to advances in diagnosing cognitive disorders and the increased awareness among the public and health professionals, patients with various forms of dementia are increasingly getting diagnosed in the mild stage of the diseases. Therefore, it is pertinent to gain knowledge of the factors affecting the outcome of patients with mild dementia.

## 2. Aim of the Study

The aim of our study was to examine the prevalence of depressive symptoms among newly diagnosed home-dwelling patients with mild dementia and the effect of depressive symptoms on all-cause mortality of patients with mild dementia over a three-year follow-up period.

## 3. Method

This study is a post hoc analysis based on the Danish Alzheimer's Intervention Study (DAISY) data. The main objective of the DAISY study was to examine the long-term efficacy of an early psychosocial counselling and support programme for home-dwelling patients with mild dementia and their caregivers. Detailed information regarding the DAISY study design, methodology, and its interventions is published elsewhere [27].

In brief, 330 newly diagnosed home-dwelling patients with mild dementia were included in the multicenter, rater-blinded, randomized DAISY study. Psychosocial interventions including counselling, education, and support were given to the patients and their caregivers over a period of 12 months. The follow-up period of the DAISY study was three years.

### 3.1. The Study Participants

The inclusion criteria for study participation were as follows: (1) age ≥ 50 years; (2) home-living patients who were clinically diagnosed with probable AD, mixed AD with vascular component, or dementia with Lewy bodies (DLB), within the past 12 months; (3) mild dementia with Mini-Mental State Examination (MMSE) score ≥ 20.

The study participants were recruited from the designated memory clinics in five districts of Denmark. The patients were referred to these clinics from their local memory clinics, family doctors, neurologists, geriatrists, or psychiatrists. If a patient was referred by the latter, then the diagnosis of dementia had to be confirmed by a specialist in the recruiting memory clinic.

All patients met the Diagnostic and Statistical Manual of Mental Disorder (fourth edition, DSM-IV) criteria for dementia, National Institute of Neurological and Communicative Disorders and Stroke and the Alzheimer's Disease and Related Disorders Association (NINCDS-ADRDA) criteria for probable AD, or the McKeith criteria for DLB [28–30]. Patients were classified as mixed AD if they had probable AD together with vascular changes in cranial CT, which may contribute to their symptoms.

Patients with severe somatic or psychiatric comorbidities including impaired vision and/or hearing that would potentially have effects on their cooperation with the intervention program were ineligible for the study. Patients participating in other intervention studies and/or living in a nursing home at baseline were also excluded.

### 3.2. Depressive Symptoms Assessment

The Cornell Scale for Depression in Dementia (CSDD) was used to assess the depressive symptoms of the eligible participants at baseline [31]. CSDD was developed by Alexopoulos et al. in 1988. It has 19 items rating the severity of specific depressive symptoms on a scale from 0 (no problems) to 2 (severe problems). The total score ranges from 0 to 38, with higher score indicating more depressive symptoms.

For the present study, based on the interviews with both patients and their caregivers, the interviewing clinicians rated the patients' depressive symptoms using CSDD [32]. A Cornell score of 0–7 indicates a patient without or with few depressive symptoms, a score of 8–10 indicates moderate depressive symptoms, and a score of >10 indicates major depressive symptoms (MD-S) [31].

### 3.3. Mortality

We obtained all-cause mortality data for the study population from the Danish Civil Registration System by using the unique identification (CPR) number assigned to every resident in Denmark [33].

### 3.4. Covariates

The patients' demographic, lifestyle, and clinical characteristics were obtained at baseline from either registers or self-reported information.

A patient's demographic information included, beyond age and sex, also household status as living alone or not. We used education as a proxy for socioeconomic status. Our study population were generally well educated; they all had high school education; therefore we counted the number of years of the education on top of the high school education and grouped the data as short education (<3 years), medium education (3-4 years), and long education (>4 years).

Lifestyle variables were self-reported including smoking status (current, ex-smoker, and never smoked) and alcohol consumption (no alcohol, ≤1 unit/day, 2-3 units/day, and >3 units/day). Body weight and height were also self-reported. Body mass index (BMI = weight in kilograms divided by the square of the height in meters, i.e., kg/m^2^) was computed and divided into three groups: normal weight (18.50 ≤ BMI < 24.99), overweight (25.00 ≤ BMI < 29.99), and obese (BMI ≥ 30.00) based on the World Health Organization standard of BMI classification [34]. Due to sample size limitation, we combined the underweight (BMI < 18.50) group with the normal weight group for the analyses. However, sensitivity analysis was conducted.

Regarding patients' clinical characteristics, we assessed the following aspects:

1. Mini-Mental State Examination (MMSE) was used to assess the cognitive status of participants [35]. The score ranges from 0 to 30 with higher scores indicating a better cognitive performance. The MMSE has both validity and reliability for the diagnosis and longitudinal assessment of dementia. All patients referred to the designated memory clinic were examined using MMSE, and a baseline MMSE score of at least 20 or above (MMSE ≥ 20) was one of the inclusion criteria for the study participation.
2. Charlson Comorbidity Index (CCI) is a weighted index that takes into account the number and the seriousness of comorbid illnesses which might affect mortality [36]. More severe diagnoses have higher weights. For every 10 years of age above 40, 1 point is added to the risk. For our study, 19 predefined diagnostic codes were used to identify comorbid illnesses recorded in the National Hospital Register for each patient during the 3 years before the dementia diagnosis, and CCI was computed based on the identified comorbidities. The scores were then grouped into three categories: 0, 1, and 2 or more.
3. The patient's awareness of their memory problem was assessed using the Anosognosia Rating Scale (Anosognosia), which is a three-point scale measuring the awareness of memory loss as “full awareness,” “shallow awareness,” and “no awareness” [37].
4. The Quality of Life Scale for Alzheimer's Disease (QoL-AD) contains 13 items and ranges from 13 to 52 with higher scores indicating a better QoL [38]. In our study, QoL-AD was rated by the primary caregiver of a patient.
5. The Alzheimer's Disease Cooperative Study Activity of Daily Living Scale (ADSC-ADL) was used to assess the activities of daily living for the Alzheimer's patients [39]. It has 23 items ranging from 0 to 78. A patient with a higher score indicated a better daily activities function.

Besides the clinical assessments, the medications for depression treatment within 12 months before the study inclusion were self-reported by the patients (yes/no).

### 3.5. Statistical Analysis

The differences in the patients' characteristics between the categories of depressive symptoms were tested using either a Kruskal-Wallis test for continuous variables or a Pearson chi-square test for categorical variables.

Cox regression models were used to evaluate the excess mortality in relation to the levels of depression severity in patients with mild dementia. Hazard ratios (HRs) and corresponding 95% confidence intervals (95% CIs) were calculated for all-cause mortality. Both crude and multivariable analyses adjusting for the potential confounding factors (age, sex, smoking, alcohol consumption, education, BMI, household status, MMSE, CCI, QoL-AD, NPIQ, ADSC-ADL, medication, and RCT allocation) were performed.

All test results with *p* < 0.05 were considered as statistically significant. All analyses were performed using SAS statistical software (version 9.4).

### 3.6. Ethical Consideration

The DAISY trial was conducted in accordance with the Helsinki Declaration. According to the Danish Act on Research Ethics, approval from the regional ethical committee was not required. However, the protocol was presented to the regional ethical committee for Copenhagen and Frederiksberg municipalities, and the committee reported that no approval was needed (ID Number (KF) 02-005/04). The Danish Data Protection Agency approved the DAISY project (ID Number 2003-41-3178), and the project was registered in the Clinical Trial Database (ISRCTN74848736). All patients and their primary caregivers gave their written consent to participate in the trial.

## 4. Results

### 4.1. The Patients' Baseline Characteristics

The mean age of the study population was 76.2 years, 151 were males, and 179 were females (Table 1). The majority of the patients had probable AD (72.4%), followed by mixed AD (24.9%) and DLB (2.7%).

At the baseline, 34 (10.3%) patients had MD-S, 54 (16.4%) had moderate depressive symptoms, and 242 (73.3%) had no or few depressive symptoms. In the MD-S group, 15 (44.1%) individuals were treated with antidepressants, which is higher than the group with moderate depressive symptoms (27.8%) and the group with no or few depressive symptoms (22.7%).

There was no statistically significant difference in sex, household status, education, smoking status, alcohol consumption, and BMI status between the three graduations of depressive symptoms. However, clinical assessments including CCI, QoL-AD, ADSC-ADL, and NPIQ were significantly different across these three groups (*p* < 0.05) (Table 1).

### 4.2. All-Cause Mortality at 3-Year Follow-Up

During the 3-year follow-up period, 56 (17.0%) patients died. Of them, 12 patients had MD-S (including 4 persons treated with antidepressants at baseline), 6 had moderate depressive symptoms, and 38 had no or few depressive symptoms at baseline.

**Figure 1:**
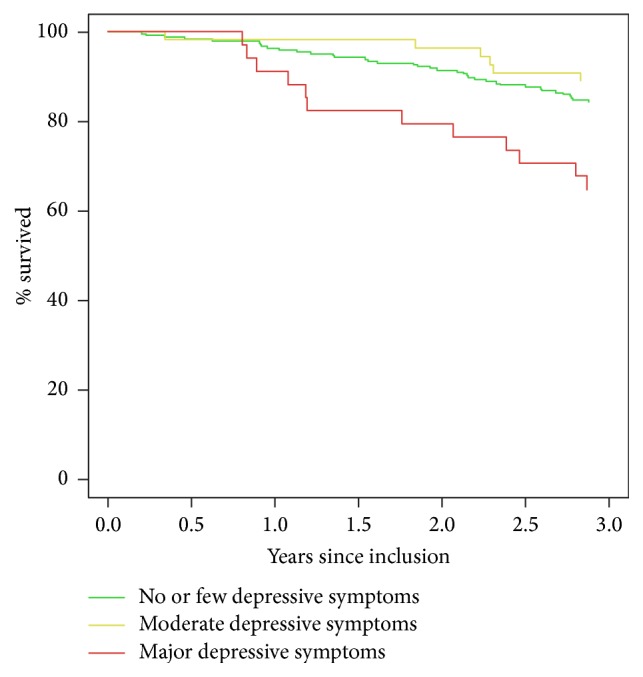
Depressive symptoms and death.

Kaplan-Meier survival curves for the three groups are presented in Figure 1. Comparison by log-rank test showed a significant difference in the mortality rate over the follow-up period only between the MD-S group and the group with no or few depressive symptoms, that is, the reference group.

Multivariable analysis adjusting for the potential confounders (model 2) showed that patients with MD-S had an almost 2-fold increased mortality compared to the reference group [HR = 2.13, 95% CI (1.05–4.34), *p* = 0.037]. Further adjusting for the clinical assessments (model 3) showed an even more increased all-cause mortality [HR = 2.50, 95% CI (1.03–6.06), *p* = 0.042] during the three-year follow-up period (Table 2). Both crude and multivariable analyses showed no statistically significant increased mortality for patients with moderate depressive symptoms compared to the patients with few or none depressive symptoms (Table 2).

The interaction between the depressive symptoms and dementia severity represented by the MMSE score was insignificant. The interaction between the depressive symptoms and the intervention between the randomized and the control group was also insignificant. A sensitivity analysis where the BMI groups were expanded to including an underweight group (BMI < 18.50) did not change the study conclusions.

## 5. Discussion

In this post hoc analysis of 330 home-dwelling patients newly diagnosed with mild dementia, we found that the patients with MD-S had significantly higher all-cause mortality compared to the patients without or with few depressive symptoms during the three-year follow-up period. The patients with moderate depressive symptoms did not have increased all-cause mortality as compared to the reference group.

There is not much evidence of the association between depressive symptoms and mortality in home-dwelling patients with dementia and even less evidence for patients newly diagnosed with mild dementia. Of the existing studies, differences in the study samples, study design, follow-up period, potential confounders, and measurement of depressive symptoms make them difficult to compare.

To the best of our knowledge, the study that is closely comparable to our study is by Lara et al., a recently published community-based study among individuals aged ≥ 65 years exploring the excess mortality due to depressive symptoms. They found that, among newly diagnosed AD patients (i.e., incidence of AD patients during 5.3 years of follow-up), depressive symptoms (measured in CES-D) were associated with higher mortality [HR = 1.88, 95% CI (1.12–3.18)] compared to no depressive symptoms [40].

However, neither Lara et al.'s nor our study assessed the presence of depression based on clinical criteria. Although 44% of patients with MD-S in our study were also treated with antidepressants, we do not know whether they had a diagnosis of major depression. Furthermore, we cannot identify whether some patients might have had major depression but they were treated with antidepressants and therefore might not present any depressive symptoms at the time we included. However, we additionally performed analysis among patients with antidepressants treatment at baseline, although an increased mortality risk was shown among patients with MD-S compared to the reference group in model 3, but due to the sample size limitation, we cannot provide a concrete conclusion. Among those without antidepressants treatment at baseline, no significant mortality risk was observed across different severity of depressive symptoms (data not shown in this article but are available for request).

Nevertheless, despite the differences in methodologies, our finding is generally in line with previous studies that also found high all-cause mortality among individuals with concurrent depression and dementia [22, 25, 41–43]. A study conducted by Janzing et al. studying the relationship between depression and mortality in elderly subjects with mild dementia living in nursing homes found that individuals who also had moderate to severe depression (Geriatric Mental State) were at higher risk of death during 1-year period than individuals without such depression [OR = 4.31, 95% CI (1.38–13.46)] [41].

Another recently published cohort study with 676 community-dwelling elderly individuals in rural Greece showed that people with mild to moderate cognitive impairment with coexisting severe depression (Geriatric Depression Scale (GDS) > 10) had increased all-cause mortality [HR = 1.77, 95% CI (1.13–2.78)] during an 8-year period [25].

Notably, the two studies mentioned above had combined moderate depression with severe depression for the mortality risk estimation. In our study, we used three different cut-offs to group the severity of depressive symptoms into three different levels, and we did not find significantly increased mortality in patients with moderate depressive symptoms compared to those with no or few depressive symptoms.

Methodological differences in ascertaining depression severity could play a role. We used an instrument that was specially developed and validated for people with dementia, the CSDD, to assess depressive symptoms in our study population [31]. The instruments used in previous studies, GDS and GMS, were often used in geriatric research but not specially developed for patients with dementia. Nevertheless, increased all-cause mortality has been consistently observed among patients with mild AD/dementia and severe depression/depressive symptoms compared to those without, regardless of the instruments used for assessing depression, speaking in favour of a true association.

An large longitudinal study by White et al. (*n* = 9560) found that the duration of depressive symptoms was associated with mortality in a dose-response manner, meaning that the longer the period with depressive symptoms a person had suffered, the higher the mortality [44]. Although 44% of the patients in our study with MD-S reported treatment with antidepressants within the 12 months before the baseline mesurement, we lack further information on how long the patients had had depressive symptoms prior to baseline, and therefore we could not confirm the aforementioned findings of White et al.

The present study used post hoc data from the DAISY intervention study. It may be argued that the individuals who underwent the intervention programmes had different mortality rate from the control group. Out of this concern, we added the intervention allocation as one of the potential confounders and adjusted for it in the final model. We also performed an interaction test and found no significant interaction between the intervention and the depressive symptoms for 3-year mortality risk; that is, the depressive symptom effect is not different between the randomized and the control group (data not shown in this article but are available for request).

Although we found that the patients with MD-S had significantly higher all-cause mortality compared to the patients without or with few depressive symptoms during the three-year follow-up period, because of the relatively small effect size of our study, we cannot be sure that we have not missed clinically relevant effects, but we are sure that (with the usual 5% error = significance level) significant effects are not chance findings. Nevertheless, besides mortality, depression indirectly affects the quality of life of the patients and their families [45, 46] and therefore must be detected and treated early on among patients with dementia.

Despite the limitations, our study has some advantages. We carefully adjusted our analyses for possible confounding through the inclusion of a wide range of indicators representing socioeconomic status, lifestyle factors, and clinical characteristics, including indicators of cognitive impairment. Additionally, we also included comorbidities and the use of antidepressants.

## 6. Conclusion

Although the etiology of depression in dementia is probably multifactorial, the results of our study indicate that patients with mild dementia due to AD or DLB and concurrent with MD-S had higher mortality compared to those with no or few depressive symptoms. It is therefore important to pay special attention to this particular group of patients, given that depression can be effectively treated.

## Figures and Tables

**Table 1:** Baseline characteristics of patients with mild dementia stratified by Cornell Scores.

Variables	Total (*n* = 330)	Category	Cornell score 0–7 (*n* = 242)	Cornell score 8–10 (*n* = 54)	Cornell score >10 (*n* = 34)	Test (*p* value)
Age, median (interquartile range)	77.1 (9.4)		77.1 (9.1)	75.7 (9.8)	78.6 (12.4)	0.15

Sex	151	Male	112	21	18	0.41
	179	Female	130	33	16	

Household status	102	Living alone	78	14	10	0.65
	228	Living with others	164	40	24	

Education	205	Short education (<3 yrs)	151	34	20	0.10
	90	Medium education (3-4 yrs)	70	14	6	
	35	Long education (>4 yrs)	21	6	8	

Smoking status	95	Never smoked	70	15	10	0.99
	147	Past smoker	108	25	14	
	88	Current smoker	64	14	10	

Alcohol consumption	26	No alcohol	20	2	4	0.20
	235	≤1 unit per day	171	39	25	
	57	2-3 units per day	45	8	4	
	12	>3 units per day	6	5	1	

BMI	172	Normal	124	29	19	0.41
	118	Overweight	88	21	9	
	39	Obese	30	3	6	

MMSE, median (interquartile range)	24.0 (4.0)		24.0 (4.0)	24.0 (5.0)	24.0 (4.0)	0.76

Dementia format	239	Probable AD	175	43	21	0.12
	82	Mixed AD	62	10	10	
	9	DLB	5	1	3	

CCI	137	0	110	14	13	0.01
	140	1	90	34	16	
	53	2	42	6	5	

Anosognosia	35	No awareness	28	5	2	0.66
	196	Shallow awareness	139	36	21	
	98	Full awareness	75	13	10	

Treated with antidepressants within 12 months	85	Yes	55	15	15	0.02
	239	No	183	38	18	

QoL-AD, median (interquartile range)	33.0 (8.0)		35.0 (8.0)	30.0 (7.0)	29.0 (8.0)	<0.0001

ADSC-ADL, median (interquartile range)	64.0 (16.0)		65.0 (16.0)	62.5 (13.0)	61.0 (15.0)	0.04

NPIQ, median (interquartile range)	3.0 (4.0)		2.0 (3.0)	6.0 (6.0)	7.0 (7.0)	<0.0001

Randomisation	163	Control	110	32	21	0.06
	167	DAISY intervention	132	22	13	

MMSE = Mini-Mental State Examination.

Probable AD = probable Alzheimer's disease.

Mixed AD = mixed Alzheimer's disease with vascular component.

DLB = dementia with Lewy bodies.

QoL-AD = Quality of Life Scale for Alzheimer's Disease.

ADSC-ADL = Alzheimer's Disease Cooperative Study Activities of Daily Living Scale.

CCI = Charlson Comorbidity Index.

*p* values are from a Kruskal-Wallis test (continuous variables) or a Pearson chi-squared test (categorical variables).

**Table 2:** The association of depressive symptoms with mortality in patients with mild dementia.

	Number of deaths	Model 1	*p* value	Model 2	*p* value	Model 3	*p* value
		HR (95% CIs)		HR (95% CIs)		HR (95% CIs)	
No or few depressive symptoms (Cornell score of 0–7)	38	1	—	1	—	1	—
Moderate depressive symptoms (Cornell score of 8–10)	6	0.68 (0.29–1.61)	0.384	0.70 (0.28–1.75)	0.446	0.48 (0.17–1.37)	0.172
Major depressive symptoms (Cornell score of > 10)	12	2.55 (1.33–4.87)	0.005	2.13 (1.05–4.34)	0.037	2.50 (1.03–6.06)	0.042

Model 1: crude analysis without adjustment for potential confounders.

Model 2: multivariable analysis adjusted for age, sex, smoking status, alcohol consumption, education, BMI, household status, MMSE, CCI, and RCT.

Model 3: multivariable analysis adjusted for all the confounders in model 2 and Qol-AD, NPIQ, and ADSC-ADL, treatment with antidepressants within 12 months.

HR = hazard ratio. 95% CIs = 95% confidence intervals.
